# Access barriers to in vitro diagnostics in Cambodia, Indonesia, Lao PDR, and the Philippines: programmatic lessons from NEDL field missions

**DOI:** 10.1186/s41182-026-00956-0

**Published:** 2026-04-22

**Authors:** Shogo Kanamori, Norielyn M. Evangelista, Nenita G. Marayag, Richard Albert J. Ramones, Sau Sokunna, Weni Muniarti, Bouaphanh Khamphaphongphane, Youthanavanh Vonghachack, Antonio F. Dela Resma Villanueva, Manami Uechi, Yuriko Egami, Naofumi Hashimoto, Eiichi Shimizu, Masataro Norizuki, Masami Fujita

**Affiliations:** 1 Bureau of Global Health Cooperation, Japan Institute for Health Security, 1-21-2 Toyama, Shinjuku-Ku, Tokyo, 162-8655 Japan; 2 Office for Health Laboratories, Department of Health, San Lazaro Compound, Santa Cruz, 1003 Manila, Philippines; 3 Department of Hospital Services, Ministry of Health, Home No. 80, Samdech Penn Nouth Boulevard, Phnom Penh, Cambodia; 4 Office for Directorate of Primary Healthcare Governance, Ministry of Health, Gd. Dr. Adhyatma Lantai 7 Blok C, Jl. HR. Rasuna Said Block X5 Kav. 4-9, Jakarta, 12950 Indonesia; 5 National Center for Laboratory and Epidemiology, Ministry of Health, WJM8+RG8, Vientiane, Lao PDR; 6 Department of Healthcare and Rehabilitation, Ministry of Health, XJ48+FFP, Ban Thatkhao, Sisattanack District, Rue Simeuang, Vientiane, Lao PDR; 7 Health and Social Welfare Unit, Economic Research Institute for ASEAN and East Asia, 6/f Sentral Senayan II, No. 8 Jl. Asia-Afrika, Gelora Bung Karno, Senayan, Jakarta Pusat, 10270 Indonesia; 8 Independent Consultant, Flat 3, No. 4 Rose Street, Salama Park, Lusaka, Zambia

**Keywords:** Diagnostics, Access to healthcare, Low- and middle-income countries, Health systems, Health service delivery

## Abstract

**Background:**

Access to essential in vitro diagnostics (IVDs) remains limited in many low- and middle-income countries, where persistent challenges exist in financing, human resources, infrastructure, procurement, supply chain management, and health insurance arrangements. Despite growing regional initiatives, such as the Association of Southeast Asian Nations (ASEAN) Essential Diagnostics List Initiative, country-specific evidence on barriers to IVD access remains limited. This article summarizes barriers to accessing IVDs and related lessons identified from field missions conducted as part of a technical cooperation project supporting the development of the National Essential Diagnostics Lists (NEDLs) in Cambodia, Indonesia, Lao PDR, and the Philippines.

**Methods:**

Field missions were conducted in the four countries between December 2024 and May 2025. Mission reports and interview records derived from key informant interviews with government officials and health workers from ministries of health, health facilities, local health offices, and health insurance agencies were examined. Statements related to potential barriers to accessing IVDs were coded, and emergent concepts were organized into a thematic framework consisting of domains and key access barriers.

**Results:**

Seventeen barriers to IVD access were identified across six domains (resources; procurement and supply chain; equipment maintenance; documented rules and standards; health insurance coverage; and attitudes of service recipients). Resource constraints, including limited budgets, workforce shortages, and inadequate laboratory infrastructure, were widespread. Procurement and supply chain challenges—such as weak demand forecasting, limited supplier availability, and logistical constraints—frequently resulted in stockouts. An important operational insight was that delays in health insurance reimbursement disrupted supplier payments and contributed to stockouts. Additional barriers included limited equipment maintenance capacity, gaps in guidelines and regulations, insufficient insurance coverage, and demand-side constraints such as low awareness and trust in diagnostic tests.

**Conclusions:**

Access to IVDs in the four ASEAN countries is constrained by multifaceted health system barriers. This study highlights an underrecognized mechanism—insurance reimbursement delays leading to stockouts—that warrants policy attention in the Asian context. As countries progress from the NEDL development to implementation, the six-domain, 17-barrier framework offers a practical tool for identifying system bottlenecks and guiding targeted interventions to ensure equitable access to essential IVDs.

## Background

Ensuring access to essential in vitro diagnostic (IVD) tests is fundamental for achieving Universal Health Coverage in low- and middle-income countries (LMICs). Despite this, 47% of the world’s population still has little to no access to diagnostics [1]. These diagnostic gaps are especially pronounced at primary care facilities, which serve as the first point of contact for a large share of the population, particularly in underserved and rural areas [2]. Such gaps can adversely impact multiple areas, including public health, early diagnosis and treatment, infectious disease control, and chronic disease management. In practice, inadequate access to diagnostics hinders the initiation of appropriate treatment and contributes to an increased burden of disease, including illness and disability [1].

Limited access to IVDs in LMICs largely stems from insufficient resources, such as inadequate funding [3–6], shortages of trained personnel [3–10], and limited infrastructural capacity [4, 5, 10, 11]. Geographic inequalities further exacerbate these gaps, making access in remote and rural settings especially difficult [3, 7, 10]. Health system-level barriers also play a substantial role, including unstable supply chains and laboratory information systems [5, 10–13], procurement inefficiencies [4], weak capacity for maintaining laboratory equipment [14], insufficient policy frameworks and technical guidance [5, 11, 15], poorly functioning referral systems of patients [3, 7, 8, 16], and ineffective specimen transport networks [2, 10, 17–20]. On the patient side, access to IVDs is further constrained by high out-of-pocket and transportation costs [7, 8, 10], limited awareness and understanding of diagnostic services [7, 10, 16], concerns about the reliability of services [3, 5], and stigma associated with certain diseases [7, 8, 10].

To address these challenges, several global and regional initiatives have been launched. The World Health Organization (WHO), through the resolution of the 76th World Health Assembly (WHA76.5), has urged Member States to strengthen diagnostic capacity by elevating it as a national priority and implementing integrated approaches. These include reinforcing laboratory networks; applying health technology assessment for the systematic evaluation of effectiveness and cost-effectiveness of diagnostics; expanding accessible and affordable packages of essential diagnostic services at the primary care level; strengthening the training of laboratory personnel; committing resources to research and product development; leveraging international and regional collaboration to harmonize regulatory, manufacturing, and supply practices for diagnostics; and establishing routine data collection systems to monitor market shaping and effective use of diagnostics [21]. The WHO has also launched the Model Essential In Vitro Diagnostic List (EDL) to guide LMICs in developing their national EDLs (NEDL). The list is intended to help countries direct limited resources toward priority IVDs and, in turn, improve access to these essential diagnostics [22]. In the Association of Southeast Asian Nations (ASEAN) Region, the ASEAN Diagnostic Security and Self-Reliance (ADxSSR) initiative was launched in 2024 with the aim of strengthening diagnostic service delivery by expanding local manufacturing of IVDs, harmonizing regulatory frameworks, and promoting research and development [23]. In addition, the ASEAN Essential Diagnostics List Initiative, also initiated in 2024, has advocated for ASEAN Member States to develop and implement their NEDLs [23].

In line with these efforts in the ASEAN Region, the Economic Research Institute for ASEAN and East Asia (ERIA) and the Japan Institute for Health Security (JIHS), under the Ministry of Health, Labor, and Welfare of Japan, launched the partnership technical cooperation project “Initial Assessment for the Development of National Essential Diagnostics List in ASEAN Countries” in May 2024. As part of this project, field missions were conducted in Cambodia, Indonesia, Lao PDR, and the Philippines between December 2024 and May 2025. The field missions’ two main objectives were: (1) to identify the objectives of developing an NEDL within each country’s specific context, and (2) to identify a range of barriers to accessing IVDs. The first component, which focused on the objectives of NEDL development, has been published as a research article [24]. The present article is based on the second objective, examining barriers to accessing IVDs. Against this backdrop, this study summarizes stakeholders’ perspectives on potential barriers to accessing IVDs and related lessons identified from the field missions conducted as part of the technical cooperation project supporting the development of NEDL across the four ASEAN countries. It also proposes a framework to address country-specific access barriers, particularly during the NEDL implementation phase.

The four countries included in this study—Cambodia, Indonesia, Lao PDR, and the Philippines—are lower- and middle-income countries in Southeast Asia with diverse health system structures. Diagnostic capacity also varies across the four countries. In Cambodia, diagnostic services are largely concentrated in higher-level facilities, with limited laboratory capacity at primary care level, particularly in rural areas [25]. In Indonesia, although a relatively extensive laboratory network exists, significant disparities in access persist across its geographically dispersed regions [26]. In Lao PDR, diagnostic capacity remains constrained by limited infrastructure and shortages of trained laboratory personnel, especially outside major urban centers [27]. In the Philippines, while diagnostic services are more widely available, inequities in access and quality persist across regions and levels of care [28].

## Methods

This article presents a secondary analysis of programmatic data on operational challenges identified during multi-country field missions conducted as part of the aforementioned ERIA/JIHS partnership project supporting the development of NEDLs. The project team reviewed the field mission reports and corresponding interview records from key informant interviews conducted in Cambodia, Indonesia, Lao PDR, and the Philippines between December 2024 and May 2025 (unpublished data). According to the reports, the interviews were conducted using topic guides covering six categories—procurement, supply chain, laboratory equipment maintenance, quality assurance, regulatory work, and benefit packages—as well as other relevant aspects [24], and were digitally recorded. These materials documented stakeholders’ perspectives and experiences related to the implementation of diagnostic services in each country. To provide contextual understanding, the project team also reviewed relevant government guidelines, policy documents, and official reports related to laboratory services and diagnostics.

To systematically analyze the collected data, we employed thematic analysis using a qualitative content analysis approach. This process involved a detailed examination of statements and observations documented in the field mission reports and interview records, with particular attention to insights obtained from key informant interviews regarding stakeholders’ perspectives on potential barriers to accessing IVDs. Each relevant statement or observation was carefully extracted and assigned codes to represent key concepts and recurring ideas.

After completing the coding process, the study team conducted an in-depth analysis of the coded data, carefully examining the information to identify recurring patterns, relationships among concepts, and overarching trends. Through this systematic examination, the team identified key factors and insights that shaped stakeholders’ perspectives regarding access barriers to IVDs. The findings from this analysis were then synthesized and organized into a structured thematic framework, consisting of domains and key access barriers to IVDs identified across the four countries.

## Results

According to the field mission reports, interviews were conducted at a total of 61 locations across the four countries, with 1–20 stakeholders interviewed at each site. The exact number of participants at each location could not be determined, as the interviews were not conducted for research purposes and clear distinctions between participants were not always possible. In some instances, representatives from multiple offices were interviewed together, further complicating the identification and enumeration of individual participants. Interviewees included government officials and health workers from ministries of health, various levels of health facilities, provincial and district health authorities, and health insurance agencies. A consolidated list of all interviewed stakeholders is provided in Table 1.

**Table 1:** List of the interviewed stakeholders

	Cambodia	Indonesia	Lao PDR	Philippines
Health Ministry—Laboratory	X	X	X	X
Health Ministry—Regulatory	X	X	X	X
Health Ministry—Medical Store	X		X	
Health Ministry—NCD	X	X	X	X
Health Ministry—Communicable Disease	X	X	X	X
Health Ministry—Sub-national Office				X
Health Ministry—Other Offices	X	X	X	X
National Hospital	X	X	X	X
Regional/Provincial/District Hospital	X	X	X	X
Health Center	X	X	X	X
National/Sub-national Reference Laboratory	X	X	X	X
Provincial/District Health Office	X	X	X	
Health Insurance Bureau	X	X	X	X

*NCD* Noncommunicable disease

Information documented during the field missions was organized to summarize operational challenges related to access to IVDs across the four countries. A total of six domains were identified to categorize commonly reported barriers: resources; procurement and supply chain of IVDs; equipment maintenance; documented rules and standards; health insurance coverage; and attitudes of service recipients. Within these domains, 17 key access barriers were further identified to capture specific operational challenges reported by stakeholders. Countries are indicated for each key access barrier using anonymized country numbers (1–4) to facilitate cross-country comparison (Table 2).

**Table 2:** Stakeholder-identified key access barriers to IVDs categorized by domains

Domains	Key access barriers (country numbers)
Resources	– Lack of financial resources for purchasing IVDs (1, 2, 3, 4) – Insufficient assignments and limited capacity of personnel (1, 2, 3, 4) – Inadequate physical infrastructure of the laboratory (1, 3)
Procurement and supply chain of IVDs	– Complicated procurement process (1, 2) – Lack of suppliers selling IVDs at affordable prices (1, 3, 4) – Inaccurate forecasting of IVD demands (1, 2, 3, 4) – Limited capacity of the IVD inventory management system (1, 2, 4) – Delays in reimbursement from the national health insurance program (4) – Difficulties in transporting IVDs to health facilities (2, 4)
Equipment maintenance	– Lack of in-house maintenance technicians and their capacity (1, 2, 3, 4) – Difficulties in obtaining maintenance services (2)
Documented rules and standards	– Lack of national guidelines (2, 4) – Regulatory restrictions on performing diagnostic tests (3)
Health insurance coverage	– Financial burden from out-of-pocket costs for diagnostic tests (3, 4) – Inappropriate reimbursement price setting (2)
Attitudes of service recipients	– Lack of awareness among service recipients (4) – Lack of trust in laboratory test results (4)

The following section summarizes commonly reported perspectives and experiences documented during the field missions for each domain and key access barrier.

### Resources

#### Lack of financial resources for purchasing IVDs

In all four countries, a lack of financial resources at health facilities for purchasing IVDs was identified as the major barrier to accessing these diagnostics. Although facilities are required to provide diagnostic services in line with national guidelines, the budgets allocated by national and local governments, along with reimbursements from the national health insurance agency, are insufficient to meet these requirements (Countries 1, 2, 3, and 4). Stakeholders noted that the situation is particularly difficult at the provincial and primary care facilities (Countries 1, 2, 3, and 4).

#### Inadequate assignments and limited capacity of personnel

The inadequate assignments and limited capacity of health personnel, including medical laboratory technologists (MLTs) and pathologists, were identified as barriers to accessing IVDs in all four countries. Stakeholders noted the capacity gaps among MLTs, particularly in performing basic microscopy and parasitology tests (Country 3). Although institutions such as national referral laboratories offer training programs, participants often face long waits due to limited training slots (Country 3). Additionally, a representative from a health center reported that their facility is staffed by only one MLT, despite the government’s laboratory licensing standards requiring two (Country 3). As a result, the facility is unable to perform clinical chemistry tests, even though it possesses a chemistry analyzer. The shortage of pathologists is another key barrier. In two countries, fewer than 10 pathologists are available nationwide, all of whom are assigned to national hospitals (Countries 2 and 4). Consequently, diagnostic tests requiring a pathologist, including Pap smears, can only be performed at national-level facilities. No institution in those countries currently trains or develops pathologists. Moreover, a recent major government reform of laboratories streamlined and standardized services across all tiers (Country 1). However, personnel assignments have not kept pace with these standards, and services prescribed in the guidelines cannot always be provided.

#### Inadequate physical infrastructure of the laboratory

Inadequate physical infrastructure, particularly insufficient laboratory space, was identified as a bottleneck to performing diagnostic tests in compliance with required standards. In one country, many health centers were built before the establishment of the government’s licensing standards, and as a result, their facilities do not meet the current requirements (Country 3). Similarly, in another country, because a major reform of laboratories was implemented recently, the infrastructure of the existing laboratories has not kept pace with the new standards (Country 1).

### Procurement and supply chain of IVDs

#### Complicated procurement process

A complicated procurement process was identified as a barrier that causes delays in procuring IVDs and can ultimately lead to stockouts. A stakeholder mentioned that the issue is particularly challenging when procuring diagnostic test kits donated by the Global Fund, as the process follows the procurement mechanism of an international agency (Country 2). In addition, the prolonged administrative procedures at the Ministry of Health were cited as a cause of procurement delays (Country 2). Furthermore, due to the government’s policy of prioritizing domestic products, the procurement of imported IVDs—which make up a large proportion of the market—becomes complicated and time-consuming (Country 1).

#### Lack of suppliers selling IVDs at affordable prices

The absence of suppliers offering IVDs at affordable prices was identified as a major cause of IVD stockouts, and the situation is particularly critical in remote areas (Country 1). A stakeholder indicated that stockouts occur when bidding failures arise because suppliers choose not to participate—often because they intend to sell at a higher price (Country 3). The issue typically emerges when only one supplier holds the certificate of product registration. Stakeholders also reported that health facilities in remote areas face difficulties in securing suppliers or finding affordable products (Country 4).

#### Inaccurate forecasting of IVD demands

A common cause of stockouts identified by stakeholders in the four countries is inaccurate forecasting of test demand (Countries 1, 2, 3, and 4). In addition to inadequate planning, unforeseeable events such as disease outbreaks also contribute to stockouts (Country 3). Some IVDs—mostly point-of-care tests (POCTs)—are procured by the central medical store and distributed to health facilities; however, facilities occasionally fail to obtain these IVDs due to stockouts resulting from inaccurate forecasting at the central level (Country 2).

#### Limited capacity of the IVD inventory management system

An effective inventory management system is essential to ensure the constant availability of IVDs. However, health facilities in one of the interviewed countries still rely on a paper-based system, creating challenges in ensuring timely and accurate replenishments (Country 2). The high license fees associated with electronic inventory management systems, combined with a shortage of trained personnel, were identified as key factors hindering effective and sustainable inventory management (Country 4). Moreover, stockouts were linked to instances where the inventory management system did not function properly and failed to generate re-order alerts (Country 1).

#### Delays in reimbursement from the national health insurance program

Several stakeholders in one country identified delays in reimbursement from the national health insurance program as the primary cause of IVD stockouts (Country 4). Because most health facilities operate with precarious cash flow, delayed reimbursements often lead to delays in paying IVD suppliers. When such payment delays recur, suppliers may cease doing business with the facilities or deliver only partial quantities in subsequent orders, which in turn leads to stockouts of IVDs.

#### Difficulties in transporting IVDs to health facilities

In two countries, securing a budget for transporting POCTs from the central warehouse to health facilities was identified as a challenge (Countries 2 and 4).

### Equipment maintenance

#### Lack of in-house maintenance technicians and their capacity

Equipment maintenance technicians stationed at hospitals play a key role in ensuring the proper upkeep of laboratory equipment, thereby supporting the availability of diagnostic services. However, such maintenance technicians are not assigned to all hospitals, and even when they are present, their capacity is often insufficient (Countries 1, 2, 3, and 4). Consequently, the maintenance of laboratory equipment largely relies on manufacturers. In one country, there is no formal institution that trains technicians to perform laboratory equipment maintenance (Country 4).

#### Difficulties in obtaining maintenance services

A stakeholder reported that health facilities sometimes face difficulties obtaining maintenance services, particularly when equipment was provided by development partners or the central medical store, and manufacturers or their local agents are not readily accessible (Country 2).

### Documented rules and standards

#### Lack of national guidelines

The lack of national guidelines was identified as a factor limiting health facilities’ ability to offer diagnostic services. For example, official guidelines have not yet been established for opportunistic infections such as cryptococcosis and histoplasmosis in one country, restricting the number of hospitals that can perform the corresponding diagnostic tests (Country 2). Similarly, in two countries, the absence of national guidelines for cancer testing limits hospitals’ capacity to provide these tests (Countries 2 and 4).

#### Regulatory restrictions on performing diagnostic tests

In one country, health workers other than MLTs are not officially authorized to independently perform any diagnostic tests, including POCTs (Country 3). Consequently, task sharing in laboratory work is limited, constraining the provision of these tests at peripheral facilities that lack MLTs.

### Health insurance coverage

#### Financial burden from out-of-pocket costs for diagnostic tests

Some stakeholders reported that patients are often required to pay out of pocket for diagnostic tests, even when such tests are covered under the national health insurance program. In one country, if a facility cannot perform a covered diagnostic test and refers the patient to another facility, the patient is typically responsible for the cost despite insurance coverage, which may discourage them from undergoing the test (Country 3). In another country, patients are required to pay out of pocket for diagnostic tests when seeking care at a health center outside their home village (Country 4).

#### Inappropriate reimbursement price setting

Due to the inappropriate reimbursement setting under the national health insurance program, health facilities cannot afford to conduct certain essential tests. For example, in one country, the capitation-based reimbursement rate for outpatient consultations at hospitals is fixed, yet the operating cost of performing certain tests, such as HbA1c test, exceeds the reimbursement amount (Country 2). Consequently, some facilities are unable to perform the test for outpatients despite it being covered by insurance.

### Attitudes of service recipients

#### Lack of awareness among service recipients

Lack of awareness among service recipients was identified as a factor contributing to their negative attitudes toward undergoing health screening tests. Some stakeholders attributed the low coverage of tests among pregnant women—such as those for HIV/AIDS, syphilis, and viral hepatitis—to a lack of awareness about their importance among service recipients, and suggested that the government should strengthen health promotion activities (Country 4).

#### Lack of trust in laboratory test results

A stakeholder highlighted that a lack of trust in laboratory test results among service recipients serves as a barrier that discourages them from accessing IVDs (Country 4).

## Discussion

This study summarizes operational challenges related to access to IVDs identified during multi-country field missions conducted in Cambodia, Indonesia, Lao PDR, and the Philippines. Across the four countries, stakeholders consistently reported a broad and interconnected set of challenges spanning health system resources, procurement and supply chain management, equipment maintenance, regulatory frameworks, health insurance arrangements, and the attitudes of service recipients. While many of these issues have been documented in the global literature on diagnostic access, the field missions provided practical insights into how these barriers manifest in routine service delivery and interact with one another in the ASEAN region.

A recurring challenge reported by stakeholders was the limited availability of financial resources for procuring essential IVDs, particularly at provincial and lower-level health facilities. Insufficient operational budgets and limited reimbursement flows from national health insurance schemes hinder facilities’ ability to consistently procure essential tests. This aligns with previous research demonstrating the central role of financing constraints in limiting diagnostic availability in LMICs [3–6]. In particular, WHO has reported that only 3–5% of healthcare budgets are allocated to diagnostic services, despite the fact that approximately 70% of clinical decisions rely on diagnostic test results [29]. These findings underscore the need for national and local governments to prioritize improving access to diagnostics, particularly by mobilizing and allocating greater financial resources to this area.

Another operational challenge highlighted during the field missions was the role of reimbursement delays in contributing to stockouts of IVDs. Stakeholders reported that delayed payments from national health insurance programs can disrupt facilities’ ability to pay suppliers, ultimately affecting the availability of diagnostic reagents and tests. Previous studies examining POCT supply chain in LMICs, particularly in Africa, have indicated financial constraints as a key contributor to IVD stockouts [12, 30, 31]. In addition, previous studies have reported various causes of IVD stockouts in LMICs [32], including inaccurate quantification and forecasting of IVD demands [33–35], poor inventory management [36], inadequate distribution systems [37, 38], insufficient storage space [39], limited production capacity [40], and a lack of monitoring and supervision [31]. However, to our knowledge, reimbursement delays as a mechanism contributing to IVD stockouts have received relatively limited attention in the Asian context. Addressing payment delays within health insurance systems may therefore represent an important policy priority for improving the reliability of diagnostic supply chains.

Human resource shortages and limited capacity, especially of MLTs and pathologists, were consistently identified as barriers across the four countries. Stakeholders described gaps in basic microscopy and parasitology competencies among MLTs, as well as limited training opportunities. The acute lack of pathologists, with two countries (Countries 2 and 4) having fewer than 10 nationwide, restricts access to specialized diagnostic services such as cytology. These findings align with earlier analyses that emphasize the persistent shortage of qualified laboratory personnel in LMICs [3–10] and underscore the need for long-term workforce strategies, including expanded training programs and redistribution of staff across tiers of the health system.

Barriers related to procurement and supply chain management were among the most prominent issues highlighted by stakeholders. Consistent with previous studies in LMICs [5, 10–12], stockouts were attributed not only to deficiencies at the facility level but also to systemic weaknesses at central medical stores, including inaccurate demand projections for POCTs. The challenges faced in remote areas—where suppliers are scarce or unwilling to participate in tenders—reflect geographic inequities documented across LMICs [3, 7, 10]. These procurement and supply chain challenges suggest that improving access to diagnostics will require more robust and responsive forecasting systems, greater supplier engagement, and streamlined procurement processes. Although pooled procurement mechanisms are more difficult to implement for laboratory equipment and reagents than for medicines and POCTs, developing such mechanisms, particularly for underserved facilities in remote areas, could help ensure a more reliable and consistent supply of IVDs [21, 41, 42].

Regulatory restrictions on non-MLT health workers performing diagnostic tests constrain service delivery at peripheral facilities, limiting the potential role of task-sharing in expanding access. This finding is consistent with international debates on how scope-of-practice regulations can either facilitate or hinder access to essential POCTs [43, 44]. In the context of the studied country, enabling POCTs at peripheral facilities without laboratory services will require not only regulatory revision but also adequate training for non-MLTs, robust quality assurance systems, and continuous supervision [45–47].

Challenges related to health insurance coverage further shape patients’ diagnostic access. Despite insurance coverage, patients frequently incur out-of-pocket costs when tests are unavailable at their local facility or when reimbursement rates do not cover the full cost of service delivery. This aligns with existing evidence that inadequate insurance benefit design can reinforce financial barriers to care in LMICs [48–50]. In particular, the findings on capitation-based payments failing to accommodate the operating costs of essential tests, such as HbA1c, illustrate how reimbursement models may inadvertently reduce the availability of diagnostics within insured health systems, underscoring the need to explicitly include diagnostics in benefit packages.

While many of the identified barriers were shared across the four countries, some notable similarities and differences were observed. Common challenges included resource constraints, such as shortages of trained laboratory personnel and limited operational budgets, as well as procurement and supply chain issues leading to recurrent stockouts. In contrast, country-specific differences were evident in the underlying mechanisms of these barriers. For example, delays in reimbursement from national health insurance schemes were particularly prominent in some countries, whereas in others, geographic constraints and limited supplier availability played a more critical role. In addition, variations were observed in regulatory and policy environments, such as the availability of national guidelines and task-sharing policies for point-of-care testing. These findings suggest that, while a common framework is useful for identifying access barriers, tailored strategies are required to address country-specific health system contexts.

Beyond summarizing individual barriers, our findings suggest a hierarchical structure of constraints affecting access to IVDs. At the upstream level, system-wide factors such as limited health financing, delays in insurance reimbursement, and the absence of national policies and guidelines shape the overall enabling environment. These constraints, in turn, give rise to downstream operational challenges, including procurement delays, stockouts, and limited service availability at health facilities. In addition, several cross-cutting issues were identified that span multiple domains. Notably, delays in insurance reimbursement emerged as a key cross-cutting constraint, as they disrupt supplier payments, contribute to stockouts, and ultimately affect service delivery. Similarly, shortages of trained personnel and weak governance structures influence multiple aspects of the diagnostic system, from procurement planning to quality assurance. These findings highlight the importance of addressing both upstream system-level constraints and downstream operational bottlenecks through coordinated and integrated policy responses.

In line with ASEAN Essential Diagnostics List Initiative, the four countries studied are currently developing NEDLs based on WHO’s practical guidance [24, 51]. NEDLs identify priority IVDs tailored to each country’s needs and therefore serve as a key mechanism for improving access to diagnostics [22]. Nevertheless, the mere development of NEDLs cannot, on its own, resolve all access barriers. Once the development phase is completed, countries will move to the implementation and monitoring stage, during which the availability and accessibility of IVDs listed in NEDLs will require continuous assessment. At this stage, the framework comprising six domains and 17 key access barriers developed in this study could serve as a useful tool for identifying country-specific access barriers and for informing appropriate interventions to address them.

This study has several limitations. First, the findings are based primarily on perspectives shared by stakeholders and may not fully reflect situations supported by empirical evidence. Second, field missions were conducted for programmatic purposes, and only one province outside the capital region was visited in each country; therefore, the perspectives captured may not fully represent national situations. Third, as the data collection was based on a review of field mission reports and interview records, which consist of detailed notes rather than verbatim transcripts, we were unable to capture direct quotations from interviewees. Finally, the views of service recipients were not captured, as interviews were conducted exclusively with officials and health workers.

## Conclusions

This study reveals that access to IVDs in Cambodia, Indonesia, Lao PDR, and the Philippines is hindered by multifaceted health system barriers spanning resources, procurement and supply chains, equipment maintenance, governance, insurance design, and patient-related factors. An important operational insight of this study is the identification of how delays in insurance reimbursements can contribute to stockouts of essential IVDs—an underexplored mechanism in the Asian context. These findings highlight the need for national and local governments to prioritize these issues within their health sector strategies and action plans.

As countries advance NEDL development, our findings underscore that adoption alone is insufficient; effective implementation and routine monitoring are crucial. The six-domain, 17-barrier framework developed through this study provides a practical tool for diagnosing system bottlenecks and guiding targeted interventions.

## Data Availability

No datasets were generated or analysed during the current study.

## Abbreviations

ASEAN: Association of Southeast Asian Nations
EDL: Essential in vitro diagnostics list
ERIA: Economic Research Institute for ASEAN and East Asia
IVD: In vitro diagnostics
JIHS: Japan Institute for Health Security
LMIC: Low- and middle-income country
MLT: Medical laboratory technologist
NCD: Noncommunicable disease
NEDL: National Essential Diagnostics List
POCT: Point-of-care test
WHO: World Health Organization
